# Studying the Impact of Cement-Based and Geopolymer Concrete on the Proliferation of *Escherichia coli* and *Staphylococcus aureus* in Water-Related Applications

**DOI:** 10.3390/ma18112560

**Published:** 2025-05-29

**Authors:** Beata Figiela, Bożena Tyliszczak, Magdalena Bańkosz, Aleksandar Nikolov, Kinga Korniejenko

**Affiliations:** 1 Faculty of Materials Engineering and Physics, Cracow University of Technology, 37 Jana Pawła II Street, 31864 Cracow, Poland; beata.figiela1@pk.edu.pl (B.F.); bozena.tyliszczak@pk.edu.pl (B.T.); magdalena.bankosz@pk.edu.pl (M.B.); 2 Bulgarian Academy of Science (IMC-BAS), Institute of Mineralogy and Crystallography, Acad. G. Bonchev Str., bl. 107, 1113 Sofia, Bulgaria; y8sashko@yahoo.com

**Keywords:** geopolymer, concrete, microbial growth, *Escherichia coli*, *Staphylococcus aureus*

## Abstract

The main aim of this research was to synthesize the new geopolymer composite and test its antibacterial properties. The new composites are based on a geopolymer matrix, with the addition of carbon fiber, nano-silica and antibacterial nanopowder. The first stage of this research was the synthesis of geopolymer composites containing variable proportions of nano-additives and, as a reference material, cement. The next step was bacterial cultivation. Two different bacterial strains were selected, Gram-positive and Gram-negative (*Escherichia coli* and *Staphylococcus aureus*). In this stage, the agar microbiological medium is used for the evaluation of bacterial growth inhibition by cement and geopolymers. In the final stage, the growth of the colony was observed and the pH measurements were taken. The final assessment of efficiency was made by using optical microscopy and a colony counter based on the Petri dish. The test performed showed that the main mineralogical components are quartz, 55.0%, and mullite, with 42.1% of crystalline ingredients. EDS analysis shows that the main oxide component is SiO_2_, about 50.9%. The obtained results connected with bacteria growth show the growth of both types of bacteria on materials; however, after several days, the growth was inhibited. An assessment of microorganism growth inhibition by cement and geopolymers shows the better efficiency of geopolymer composites in this area for both types of colonies (Gram-positive and Gram-negative). The new element in this research was to plan the research from the point of view of its application in the water environment. The provided research can be useful for the inhibition of biofouling phenomena on marine and inland water infrastructure.

## 1. Introduction

The interest in the microbiological investigation of the materials, including this, dedicated for infrastructure applications, increased rapidly during the COVID-19 pandemic. At this time, the main point of research was materials for application in constructions exposed to microorganisms, such as bacteria, to prevent the spread of epidemics. The particular point of interest was the infrastructure in hospitals and similar infrastructure [1]. It is worth noting, however, that use in hospital infrastructure is only one of many areas where the antimicrobial properties of building materials are desirable. Other important areas are coating applications, also in the context of the means of the transport and spreading of some invasive species [2,3] or water and wastewater treatment [4,5].

The particular problem is connected with the microbially induced degradation of construction materials dedicated to water infrastructure, which significantly influences their durability [6]. In this case, the infrastructure deterioration is accelerated by the water availability and microorganisms’ presence, which cause the formation of undesirable substances on the surface of materials [7]. On the one hand, the existence of microorganisms on the surface is unwanted, but on the other hand, the materials should not be hazardous to water fauna and flora [7]. From this point of view, the most desirable behavior is the inhibition of growth on the material surface [8].

Currently, there are several theories about the antimicrobial properties of geopolymer materials. The basic one is connected with an aluminum presence. This theory bound antimicrobial properties with the aluminum phase manufactured during the material activation [9]. According to this theory, aluminum inhibits the growth of microorganisms and reduces metabolic acid production by damaging the cell membrane [9,10]. From this point of view, all geopolymers should have antimicrobial properties, which is not well confirmed in the literature. Another antimicrobial mechanism is joined with the presence of oxides, especially Fe_2_O_3_, PbO_2_, TiO_2_, in the material’s structure [11,12]. In this case, the mechanism is connected with forming reactive oxygen species that penetrate the cell membrane of microorganisms and influence the DNA geometry and metabolic routes, which causes the effect of cell death [7,11]. Additionally, the presence of metal cations, such as Pb^4+^, Fe^3+^ and Ti^4+^, can disturb the osmotic stability and restrict enzymatic activity through the reaction with the negatively charged envelopes of the microbial cells [11,13,14]. The other study suggests that the mechanism of antimicrobial activity is mixed and can be mixed and involve parallel mechanisms reactive oxygen species’ generation, cell wall degradation and DNA damage [15].

The previous investigations also confirm the effectiveness of nanoparticles in enhancing antimicrobial properties, including nano chromia [16], tungsten trioxide [17], zinc oxide [15,18], zinc ferrite [18], titanium oxide [12,19,20], zirconia [21], hematite [13], nickel oxide [1] and silver [22]. As an alternative for nanocomponents, also silver and copper forms of natural zeolite clinoptilolite have been investigated [3]. It is also worth noticing that the effectiveness of different nanoparticles can be driven by different mechanisms [23], including: destroying the cell membrane by “wrapping” bacteria through the graphene oxide sheets [24,25], releasing reactive oxygen species [26,27], an interaction with the DNA and proteins [28] or distorting the cellular membrane [29,30].

The motivation to undertake this research was the development of knowledge regarding the structure and antimicrobial properties of the produced geopolymer composites intended for use in the aquatic environment. The main planned application of geopolymer composites is infrastructure in sea and inland waters. One of the desirable features of such composites is resistance to the growth of bacteria and algae by aquatic organisms. At the same time, the material must be resistant to the effects of microorganisms, but must not be toxic to the fauna and flora of the tanks, so it should inhibit the growth of microorganisms, but not destroy them directly.

## 2. Materials and Methods

### 2.1. Sample Preparation and Material Testing

Spheroidal fly ash class F from the Skawina CHP plant (Małopolska, Poland) and coal shale from the Silesia mine (Silesia, Poland) were used to produce geopolymer discs with a diameter of 4 cm and a thickness of about 1 cm.

**Figure 1:**
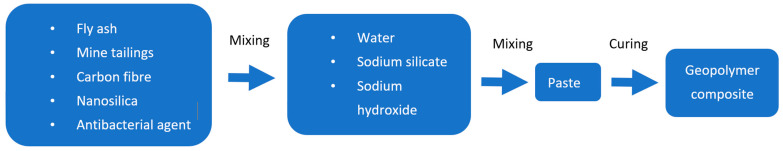
Diagram of geopolymer composite preparation.

The first stage of geopolymer production was the preparation of a 10 molar solution of a mixture of hydrated sodium hydroxide flakes with aqueous sodium silicate type R-145 (molar module 2.5, density 1.45 g/cm^3^) in a ratio of 1:2.5. Then the produced solution was left for 24 h in laboratory conditions to equalize the concentrations. After this time, it was used to produce geopolymer discs. Weighed amounts of dry materials: fly ash/material from the Silesia mine in amounts of approximately 80% by weight and approximately 20% by weight were mixed in a planetary mixer bowl (GEOLAB, Warsaw, Poland) until the particles of the components were evenly distributed throughout the volume, then the solution was added in a liquid–solid ratio (L/S) of 0.4 and mixed for about 10 min until a homogeneous mass formed. Then, 1% of 3 mm long carbon fibers were added to the mixing mass. The paste prepared in this way was placed in three sterile cups with a diameter of 4 cm to a height of 2 cm. The remaining mass was divided into three parts. To each of them, 0.5% of nanosilica with a particle size of 13–24 nm was added and mixed. To two parts of the mass, 0.5% and 1% of an antibacterial agent (98% content of Ag and 2% of a mixture of different oxides such as: P_2_O_5_, ZrO_2_, N_2_O, Y_2_O_3_, Al_2_O_3_, HfO_2_ and TiO_2_) were added, respectively. Each of the three prepared mortars was poured into sterile cups, similar to the first variant of the geopolymer mass. The samples prepared in this way were placed in a laboratory dryer at 75 °C for 24 h. The next day, they were removed from the molds and left in laboratory conditions for 28 days. The diagram for the preparation geopolymer composite is presented in Figure 1.

The last type of sample prepared in the experiment was Portland cement/sand disks in a 1:1 ratio, mixed with water and poured into sterile cups. These samples were left in laboratory conditions for 28 days until the actual test.

All the samples produced are presented in Table 1 with their corresponding weight quantities.

The main goal for particular ingredients of the geopolymer composition was connected with the following:

- Fly ash—main product for geopolymerization, an industrial by-product.
- Mine tailing (coal shale) was implemented to enhance the environment-friendly character of the composite. It is a waste from mining production and can be considered as an alternative material for fly ash.
- Carbon fiber improves the flexural strength and some works also suggest the antimicrobial properties of this material [31].
- Nanosilica—improving the compressive strength, possibly increasing the coherence of the material structure and its resistance to water penetration.
- Antibacterial agent—enhancement of antibacterial properties.

The amount of additives was defined by:

- Fly ash—the amount was dependent on the amount of other ingredients, complementing 100%.
- Mine tailing (coal shale)—the amount is based on previous research, taking into consideration the mechanical properties of the materials [32].
- Carbon fiber—the amount was determined by the workability of the mixture and the ratio between price and enhanced mechanical properties. The amount is based on the previous investigation [33].
- Nanosilica—the amount based on the literature investigation [34].
- Antibacterial agent—the amount was experimentally added as 0.5 and 1% by mass. These percentages were the results of a literature investigation [34].

For the prepared materials, the microstructure was investigated by using scanning electron microscopy (SEM), type JEOL JSN5510LV (JEOL, Tokyo, Japan), with an energy-dispersive spectrometer (EDS). The measurements were made on samples placed on carbon pots and covered by a conductive gold layer with a thickness of several angstroms using JOEL DII-29030SCT (JEOL, Tokyo, Japan). The observations were made with a voltage of 15 kV, working distance between 11 and 12 mm, and in magnifications between 100 and 2000×. Additionally, the mineralogical composition was determined using powder X-ray diffraction (XRD) measurements. These measurements were made on Empyrean (Malvern-Panalytical, Almelo, The Netherlands) diffractometer using CuKα radiation at 40 kV and 30 mA.

### 2.2. Bacteria Cultivation and Assessment of Anti-Bacterial Properties

After 28 days of seasoning, the samples were subjected to a bacterial multiplication test with *Escherichia coli* and *Staphylococcus aureus*. The following were used to conduct the entire experiment in accordance with manufacturer’s instructions:

- Fifteen material discs of five variants;
- Two sets *Escherichia coli* (Gram-negative bacteria);
- Two sets *Staphylococcus aureus* (Gram-positive bacteria);
- An agar microbiological medium is used to count many varieties of living organisms;
- Soy agar medium is used as a reference medium for the cultivation of a wide range of microorganisms;
- Chromogenic plates for selective isolation and direct identification of *S. aureus* bacteria;
- Chromogenic plates for the detection of *E. coli* bacteria after incubation;
- 0.9% NaCl solution;
- Tissue paper discs with a diameter of 1 cm, treated with a UV sterilizer.

Geopolymer discs of diameter of 4 cm were placed in sterile cups and filled with 50 mL of tap water. The cups were marked according to their content and used bacteria strain. Then, 15 min after placing in water, pH measurements were performed. The pH was measured as an average value of three readings, using a pH meter. Each measurement was preceded by immersing the meter in distilled water and then drying it. One measurement test lasted until the pH value stabilized. The cups with samples immersed in water were left for 24 h at room temperature.

In the next step, the preparation of agar microbiological medium for the evaluation of bacterial growth inhibition by cement and geopolymers was provided. Agar plates were taken out from the refrigerator and placed on a counter under UV light. Each was marked according to the markings of the cups with geopolymers: the *E. coli* set and the *S. aureus* set. The microorganisms were removed from the refrigerator, opened and placed in endorphins and 0.5 mL of NaCl was added, which activates the microorganisms. The bacteria activated in this way were applied using microbiological loops to previously prepared agar plates according to the markings. Then, they were set aside for 10 min before performing the next steps. The prepared paper discs were soaked in a cup with immersed geopolymer from the reference set and placed on the prepared substrate with the number corresponding to the cup. The procedure was repeated for each sample from the *E. coli* and *S. aureus* sets. The reference sample for both types of microorganisms was performed on tap water, designated as “0”. The plates from each set were placed one on top of the other (6 in a column), then turned 180° and placed in incubators for 24 h. After this time, bacterial multiplication was assessed. A plate with tissue paper on the border of which there was a grown colony was assessed as a lack of inhibition.

Then, the attempt to multiply *S. aureus* and *E. coli* microorganisms in containers with immersed geopolymers was conducted. The *S. aureus* microbial colony was hydrated with 0.5 mL NaCl solution and then, after a few minutes, 10 mL NaCl was added. From the test tube with hydrated and diluted bacteria, 1 mL of liquid was taken and successively added to containers marked in green with numbers 1–5 with cement and geopolymer pellets. The same procedure was repeated for the *E. coli* microbial colony. After completion, the closed cups were left for 24 h in laboratory conditions. After this time, chromogenic agar plates were prepared for selective isolation and direct identification of *S. aureus* within 18–24 h and chromogenic plates for quantitative evaluation of *E. coli* after 24 h of incubation. Cups with geopolymer material and microorganisms were shaken before inoculation. Using a microbiological loop, the liquid from the cup was applied to the inoculation medium corresponding to the designation of the type of microorganisms, indicating where the application of the material began. The process was carried out for each type of sample for the green and red colors, marked with numbers from 1 to 5. Bacteria were plated on two types of media: chromogenic for the determination of the selected bacterial strain and agar for the culturing of a wide range of microorganisms used as reference plates. The prepared plates with the inoculation of research materials were placed one on top of the other and placed in an incubator according to the time specified by the manufacturer for a given type of substrate. After removing the plates from the incubator, the growth of bacterial colonies was observed and assessed according to specifications.

The microscopic observation was made on Keyence VHX-7000 (Keyence International, Mechelen, Belgium).

## 3. Results

### 3.1. Geopolymer Investigations

**Figure 2:**
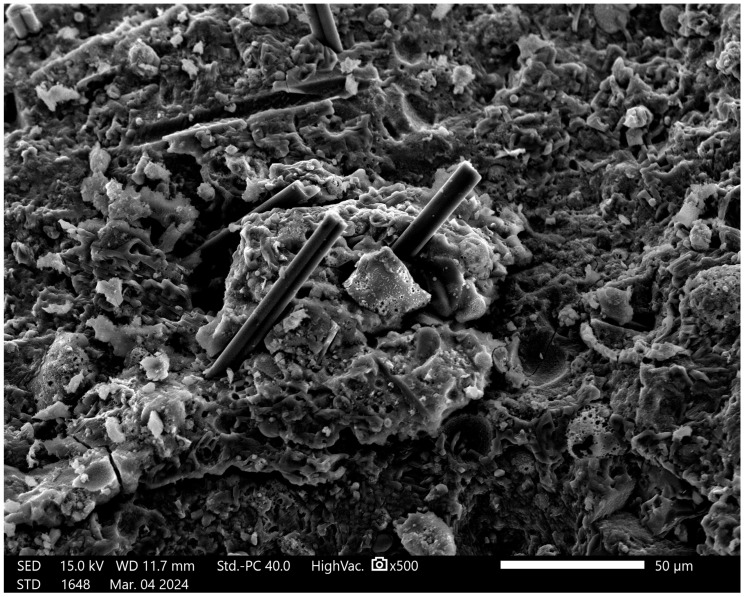
SEM image of geopolymer with 1% CF, 0.5% nanosilica and 1% antibacterial agent. Designation 5 from Table 1. Magnification is 500×.

The first step was the characterization of geopolymers after synthesis. The research results are presented on the composition of the geopolymer with 1% CF, 0.5% nanosilica and 1% antibacterial agent. The microstructure of the obtained material is presented in Figure 2.

In Figure 2, there are visible geopolymer materials with carbon fibers. The structure of the materials is typical for geopolymer composites [35]. The fiber addition is well visible and they are evenly distributed in the whole volume of the material. The nanoadditives are not visible in the matrix, probably because of their size and coherence with the matrix material. Additionally, for the presented figure, the EDS analysis under the area was provided, Table 2.

The EDS analysis shows a typical oxide composition for the geopolymer materials [36]. The main components are alumina and silica, which are typical for a geopolymer matrix. The relative amount of sodium is visible, which is an effect of the used activators—sodium silicate and sodium hydroxide. There is also a lower amount of some components that occur in fly ash and mine tailings, such as magnesium oxide, potassium oxide, sulphur oxide and calcium oxide.

**Figure 3:**
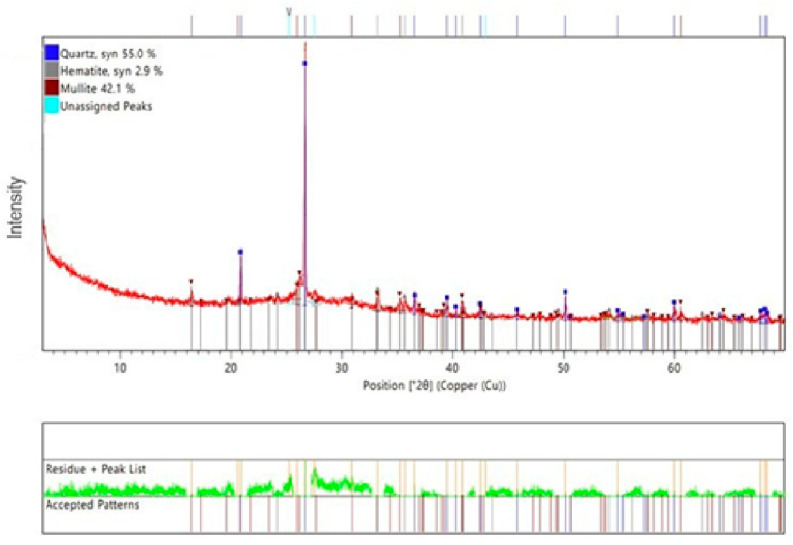
XRD patterns geopolymer with 1% CF, 0.5% nanosilica and 1% antibacterial agent. Designation 5 from Table 1.

To supplement the information about the obtained materials, the mineralogical analysis was provided, Figure 3.

The mineralogical composition shows the main components, quartz and mullite. It has been identified based on the HighScore Plus software (v5.1) and the PDF4 + crystallographic database charts no. 01-087-2096 and 01-073-2234, respectively. These two minerals commonly appear in geopolymer composites [37]. There was also an identified hematite based on the PDF software chart no. 05-001-0663. The analysis reveals the presence of a small amount of hematite, which is possibly present because fly ash and mine tailings can include some iron. This element was not visible on EDS analysis, but it has to be stressed that EDS is a qualitative investigation, not a quantitative one, and in the analyzed area, this oxide cannot be observed.

### 3.2. pH Measurement

The results of the pH measurements are presented in Table 3. Sample “0” represents the reference measurements for the water.

Measurements of the hydrogen ion concentration in water with the immersed samples showed an alkaline reaction of the solutions. The highest alkalinity was found in samples with the addition of an antibacterial agent; with the increase in the content of the geopolymer composition, the concentration of ions increased OH^−^. The high alkalinity is a common phenomenon in the case of cementitious materials, including geopolymers [38]. The practical problem in this case is connected with the time necessary for the stabilization of the material in case of underwater applications, such as artificial reefs [39]. The previous research shows that the infrastructure made from concrete or geopolymers gains a neutral pH on the surface after around 6 months [40].

### 3.3. Assessment of Microorganism Growth Inhibition by Cement and Geopolymer

#### 3.3.1. *Escherichia coli* Microorganism

**Figure 4:**
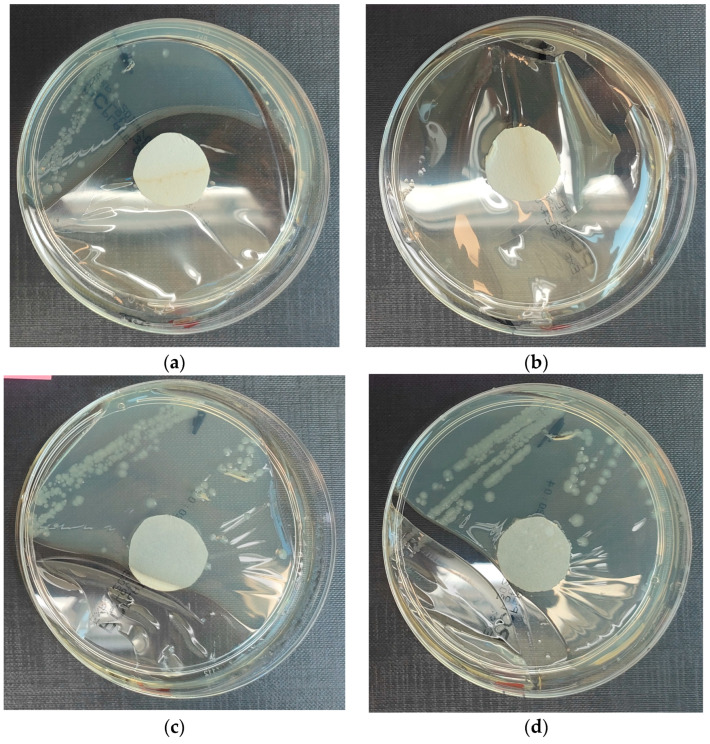

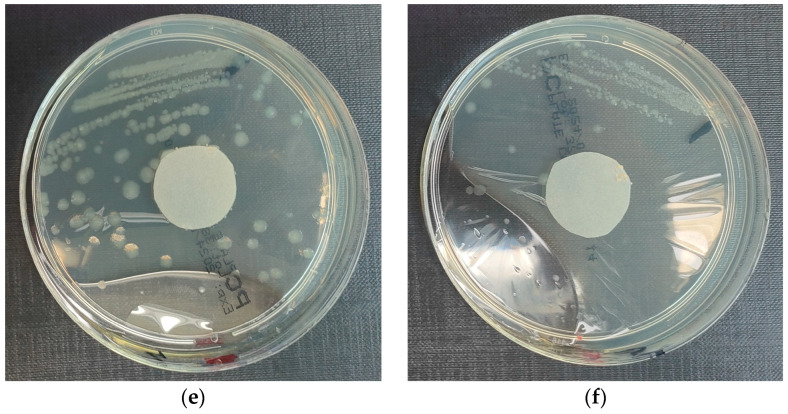
Investigation of microorganism growth inhibition using *Escherichia coli* microorganism: (**a**) Reference sample—water; (**b**) Sample 1—cement; (**c**) Sample 2—geopolymer with 1% CF; (**d**) Sample 3—geopolymer with 1% CF and 0.5% nanosilica; (**e**) Sample 4—geopolymer with 1% CF, 0.5% nanosilica and 0.5% antibacterial agent; (**f**) Sample 5—geopolymer with 1% CF, 0.5% nanosilica and 1% antibacterial agent.

The results of the bacterial growth are presented in Figure 4. The following are the specifications of the plates used for the bacteria:

- Results after incubation at 28–32 °C for 69–75 h.
- Positive controls’ inoculum of 50–120 colony-forming units:
  ○ *Bacillus subtilis* ATCC^®^ 6633 (WDCM 00003)—irregular straw colonies.
  ○ *E. coli* ATCC^®^ 25922 (WDCM 00013)—straw colonies.
  ○ *E. coli* ATCC^®^ 8739 (WDCM 00012)—straw colonies.
  ○ *S. aureus* ATCC^®^ 25923 (WDCM 00034)—cream/straw colonies.
- Colony counts shall be equal to or greater than 70% of the control medium (Tryptone Soya Agar).

An analysis of the inhibition of the growth of colonies of microorganisms from the *E. coli* group for the reference sample—water—clearly shows the growth of colonies on the surface of the paper, which means that water is a good carrier of bacteria. Microorganisms take on a beige color, forming in accordance with the movement of the inoculation. It can be observed that the growth of bacteria was not inhibited in the case of any of the samples, while for samples with geopolymers, microorganisms are located on the border of the paper immersed in the liquid with the samples. In the case of concrete, the paper is observed to be occupied by the colony on 2/3 of the surface.

#### 3.3.2. *Staphylococcus aureus* Microorganism

**Figure 5:**
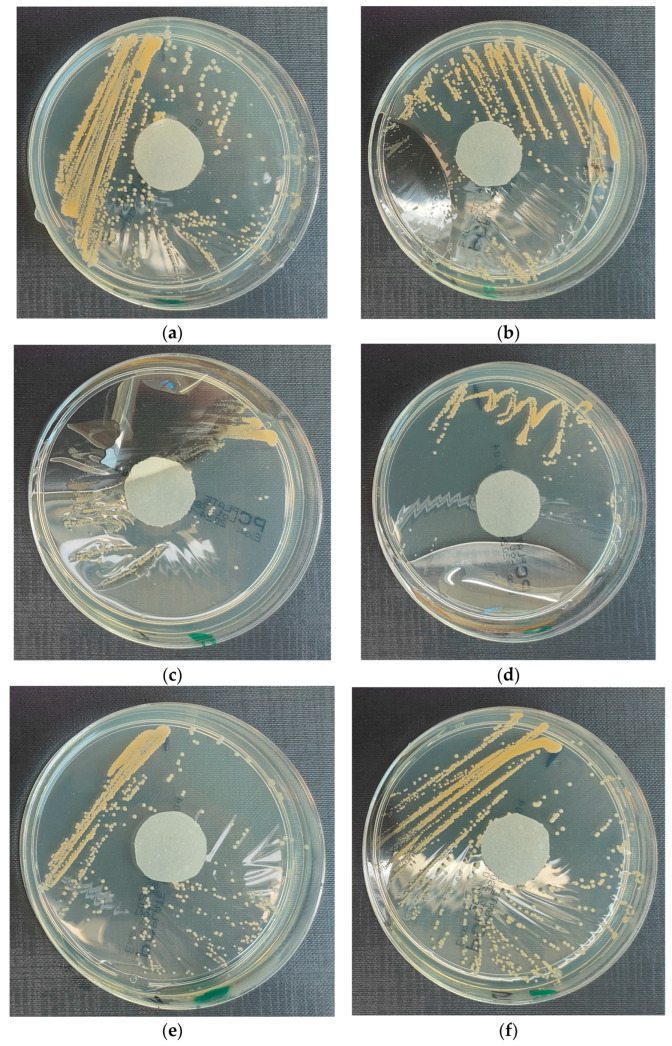
Investigation of microorganism growth inhibition using *Staphylococcus aureus* microorganism: (**a**) Reference sample—water; (**b**) Sample 1—cement; (**c**) Sample 2—geopolymer with 1% CF; (**d**) Sample 3—geopolymer with 1% CF and 0.5% nanosilica; (**e**) Sample 4—geopolymer with 1% CF, 0.5% nanosilica and 0.5% antibacterial agent; (**f**) Sample 5—geopolymer with 1% CF, 0.5% nanosilica and 1% antibacterial agent.

The results of the bacterial growth are presented in Figure 5. The following are the specifications of the plates used for the bacteria:

- Results after incubation at 28–32 °C for 69–75 h.
- Positive controls of inoculum of 50–120 colony-forming units:
  ○ *Bacillus subtilis* ATCC^®^ 6633 (WDCM 00003)—irregular straw colonies.
  ○ *E. coli* ATCC^®^ 25922 (WDCM 00013)—straw colonies.
  ○ *E. coli* ATCC^®^ 8739 (WDCM 00012)—straw colonies.
  ○ *S. aureus* ATCC^®^ 25923 (WDCM 00034)—cream/straw colonies.
- Colony counts shall be equal to or greater than 70% of the control medium (Tryptone Soya Agar).

*S. aureus* microorganisms form dense cream/straw colonies. The colony grows on the border of samples 0, 1 and 2. However, they cover the entire surface of papers immersed in liquids no. 4 and 5. Interestingly, no such effect is observed for sample no. 3.

### 3.4. Evaluation of Microbial Multiplication in Liquid with Cement and Geopolymer Samples

#### 3.4.1. *Escherichia coli* on Reference Plates

**Figure 6:**
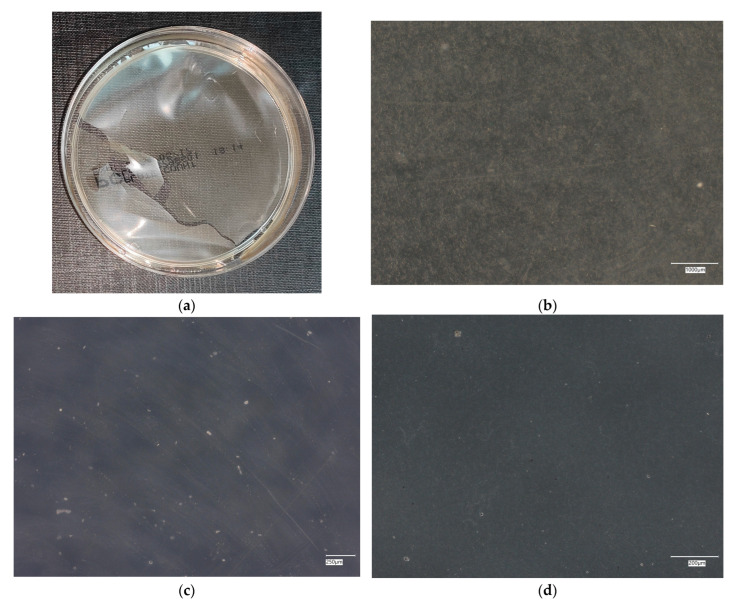
*Escherichia coli* on reference plates on sample 1—cement material: (**a**) Overall photo; (**b**) Optical microscope image under magnification 40×; (**c**) Optical microscope image under magnification 100×; (**d**) Optical microscope image under magnification 200×.

**Figure 7:**
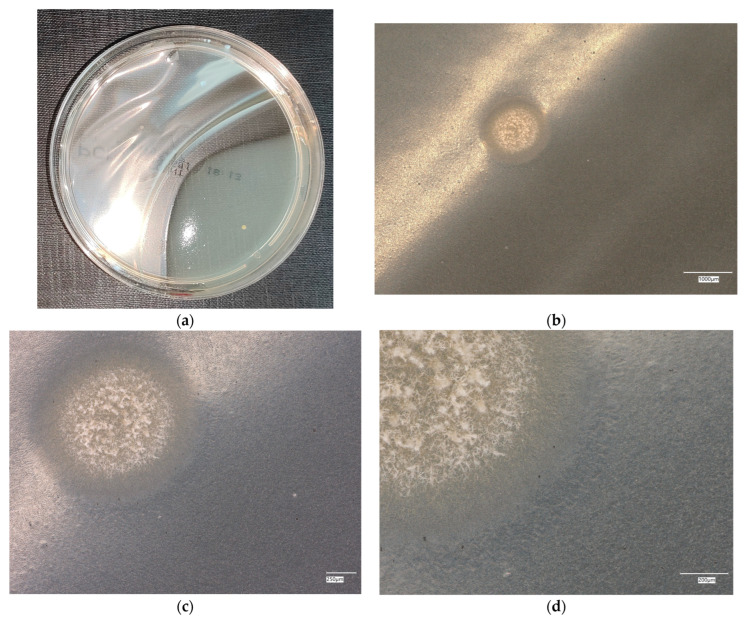
*Escherichia coli* on reference plates on sample 2—geopolymer with 1% CF: (**a**) Overall photo; (**b**) Optical microscope image under magnification 40×; (**c**) Optical microscope image under magnification 100×; (**d**) Optical microscope image under magnification 200×.

**Figure 8:**
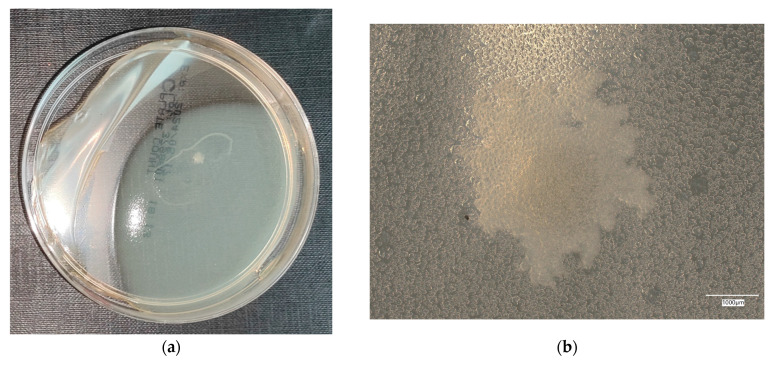

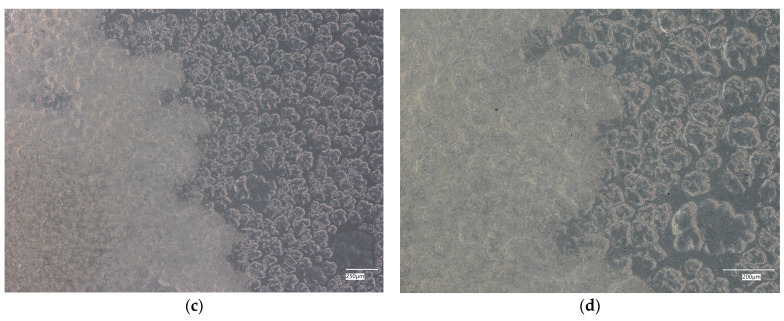
*Escherichia coli* on reference plates on sample 3—geopolymer with 1% CF and 0.5% nanosilica: (**a**) Overall photo; (**b**) Optical microscope image under magnification 40×; (**c**) Optical microscope image under magnification 100×; (**d**) Optical microscope image under magnification 200×.

**Figure 9:**
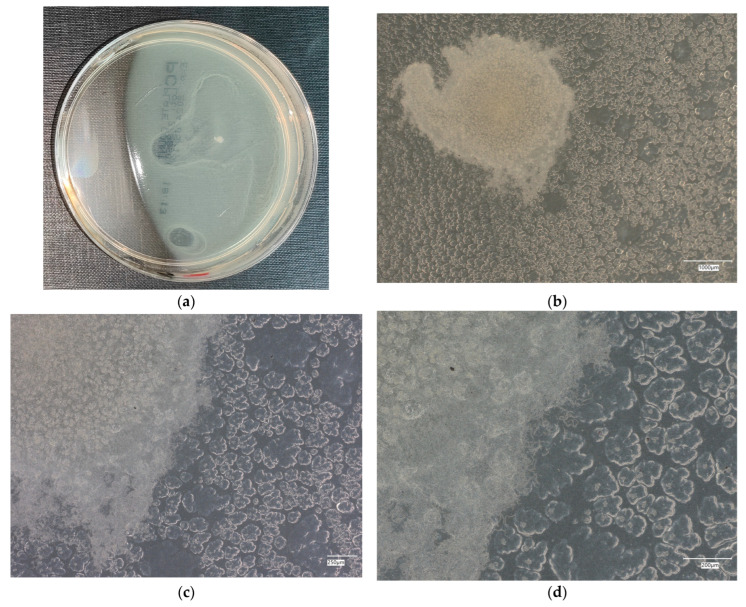
*Escherichia coli* on reference plates on sample 4—geopolymer with 1% CF, 0.5% nanosilica and 0.5% antibacterial agent: (**a**) Overall photo; (**b**) Optical microscope image under magnification 40×; (**c**) Optical microscope image under magnification 100×; (**d**) Optical microscope image under magnification 200×.

**Figure 10:**
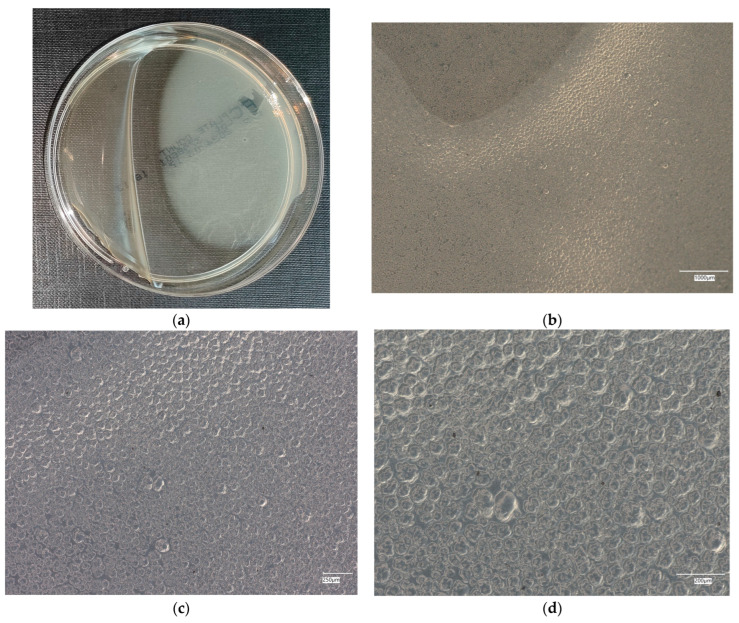
*Escherichia coli* on sample 5—geopolymer with 1% CF, 0.5% nanosilica and 1% antibacterial agent: (**a**) Overall photo; (**b**) Optical microscope image under magnification 40×; (**c**) Optical microscope image under magnification 100×; (**d**) Optical microscope image under magnification 200×.

The results of the bacterial growth are presented in Figure 6, Figure 7, Figure 8, Figure 9 and Figure 10. The following are the specifications of the plates used for the bacteria:

- Results after incubation at 28–32 °C for 69–75 h.
- Positive controls of inoculum of 50–120 colony-forming units:
  ○ *Bacillus subtilis* ATCC^®^ 6633 (WDCM 00003)—irregular straw colonies.
  ○ *E. coli* ATCC^®^ 25922 (WDCM 00013)—straw colonies.
  ○ *E. coli* ATCC^®^ 8739 (WDCM 00012)—straw colonies.
  ○ *S. aureus* ATCC^®^ 25923 (WDCM 00034)—cream/straw colonies.
- Colony counts shall be equal to or greater than 70% of the control medium (Tryptone Soya Agar).

An analysis of the results of the *E. coli* microorganism culture after incubation on the reference plates shows the cultured irregular clusters of the cream-colored colonies. For sample no. 1 (concrete), several microbial inclusions were observed, visible at a 100× magnification. For samples no. 2, 3, 4 and 5, distinct clusters of bacteria were observed in the form of spots.

#### 3.4.2. *Escherichia coli* on Chromogenic Plates

**Figure 11:**
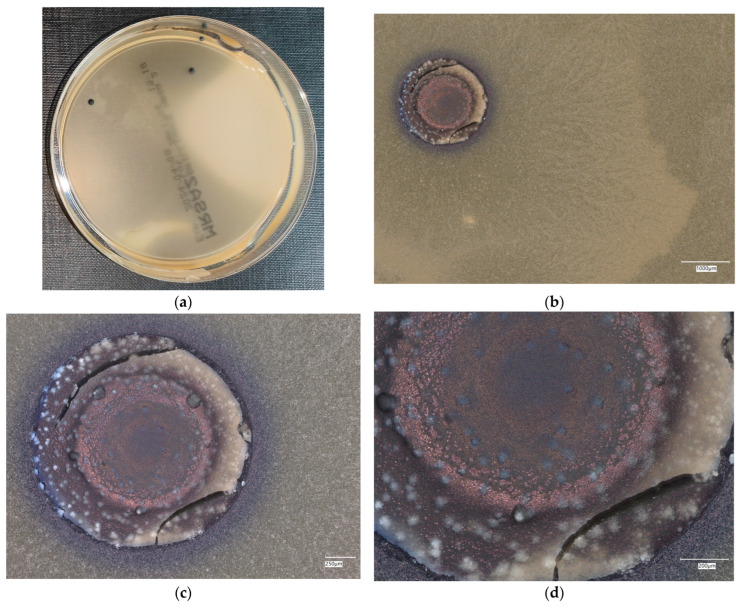
*Escherichia coli* on chromogenic plates on sample 1—cement material: (**a**) Overall photo; (**b**) Optical microscope image under magnification 40×; (**c**) Optical microscope image under magnification 100×; (**d**) Optical microscope image under magnification 200×.

**Figure 12:**
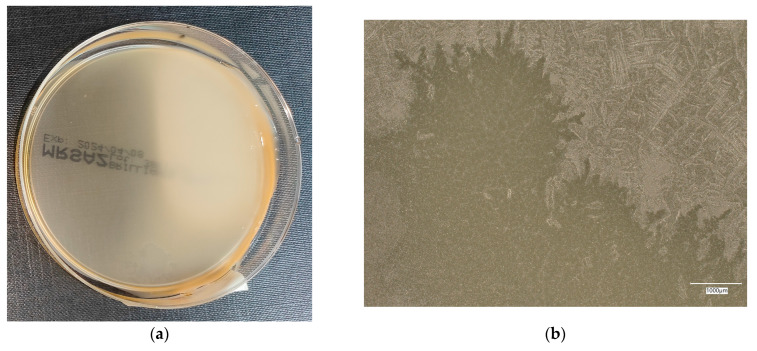

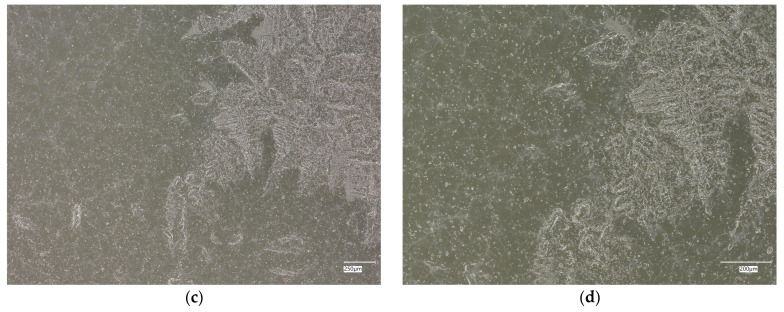
*Escherichia coli* on chromogenic plates on sample 2—geopolymer with 1% CF: (**a**) Overall photo; (**b**) Optical microscope image under magnification 40×; (**c**) Optical microscope image under magnification 100×; (**d**) Optical microscope image under magnification 200×.

**Figure 13:**
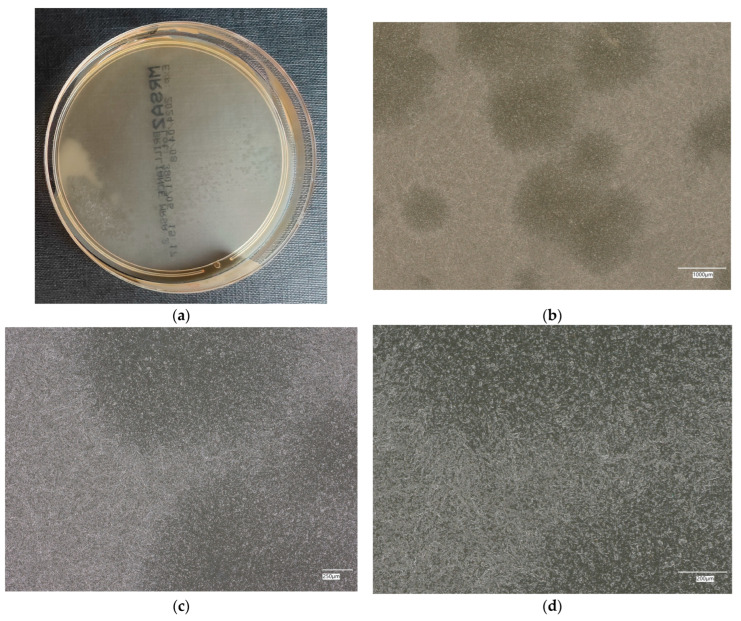
*Escherichia coli* on chromogenic plates on sample 3—geopolymer with 1% CF and 0.5% nanosilica: (**a**) Overall photo; (**b**) Optical microscope image under magnification 40×; (**c**) Optical microscope image under magnification 100×; (**d**) Optical microscope image under magnification 200×.

**Figure 14:**
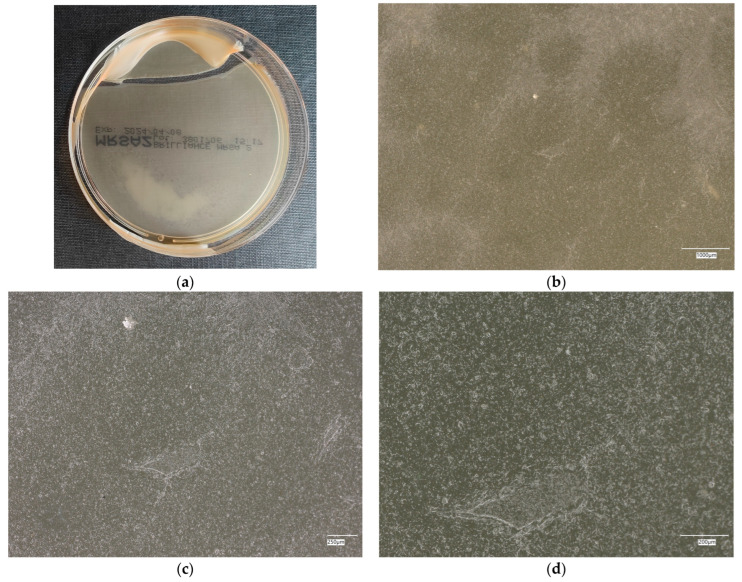
*Escherichia coli* on chromogenic plates on sample 4—geopolymer with 1% CF, 0.5% nanosilica and 0.5% antibacterial agent: (**a**) Overall photo; (**b**) Optical microscope image under magnification 40×; (**c**) Optical microscope image under magnification 100×; (**d**) Optical microscope image under magnification 200×.

**Figure 15:**
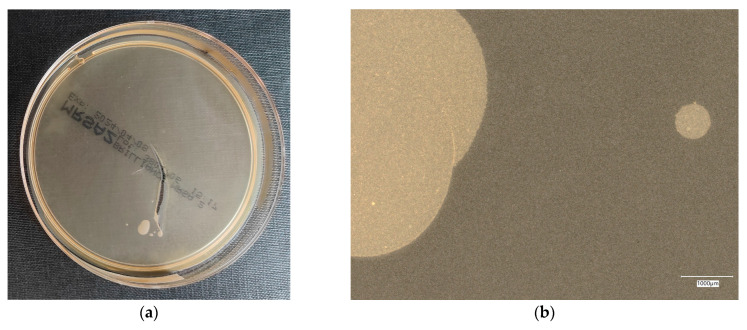

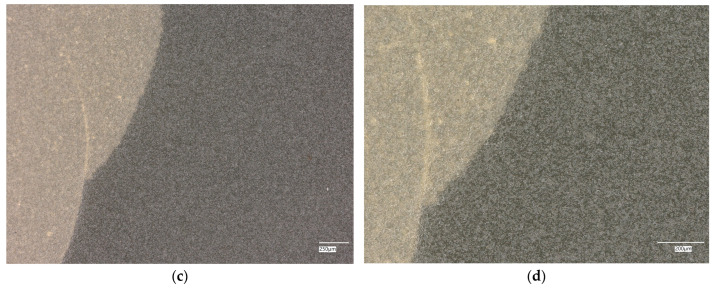
*Escherichia coli* on chromogenic plates on sample 5—geopolymer with 1% CF, 0.5% nanosilica and 1% antibacterial agent: (**a**) Overall photo; (**b**) Optical microscope image under magnification 40×; (**c**) Optical microscope image under magnification 100×; (**d**) Optical microscope image under magnification 200×.

The results of the bacterial growth are presented in Figure 11, Figure 12, Figure 13, Figure 14 and Figure 15. The following are the specifications of the plates used for the bacteria:

- Microbiological control of positive controls growth inoculum of 50–120 colony-forming units (cfu).
- Incubation conditions: 18–24 h @ 36 ± 2 °C, aerobic.
- Strains tested by membrane filtration.
- *E. coli* ATCC^®^ 8739™ (WDCM 00012) dark blue to violet colonies.
- *Enterobacter aerogenes* ATCC^®^ 13048™ (WDCM 00175) pink to red colonies.
- Colony counts shall be ≥70% of the control medium TSA.

Microbiological control after the incubation of *E. coli* colonies on a chromogenic plate showed a clear presence of bacteria on plate no. 1, taking the form of a purple pellet with shades of blue color. On other control plates, the appearance of bright areas of colony-forming units was observed, with the indication that with increasing additives in the geopolymer, the amount of this area decreases.

#### 3.4.3. *Staphylococcus aureus* on Reference Plates

**Figure 16:**
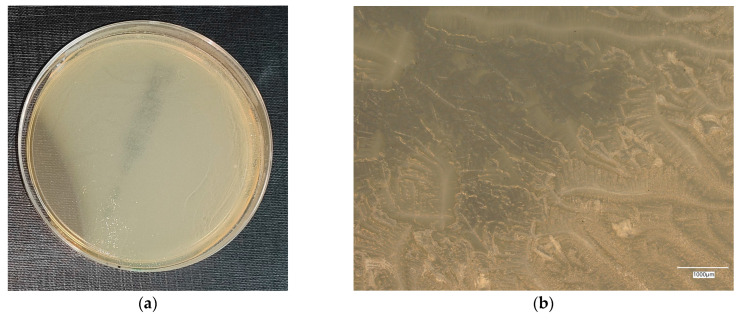

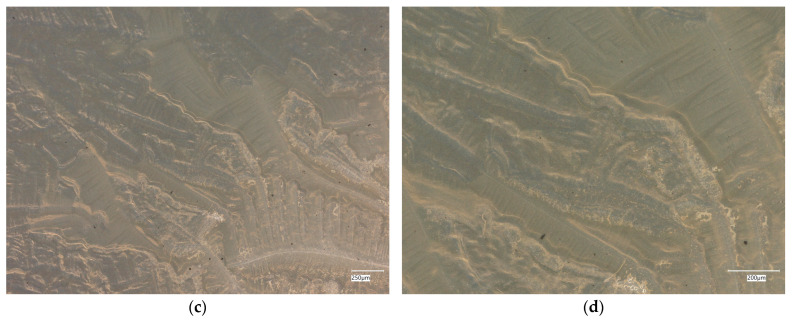
*Staphylococcus aureus* on reference plates on sample 1—cement material: (**a**) Overall photo; (**b**) Optical microscope image under magnification 40×; (**c**) Optical microscope image under magnification 100×; (**d**) Optical microscope image under magnification 200×.

**Figure 17:**
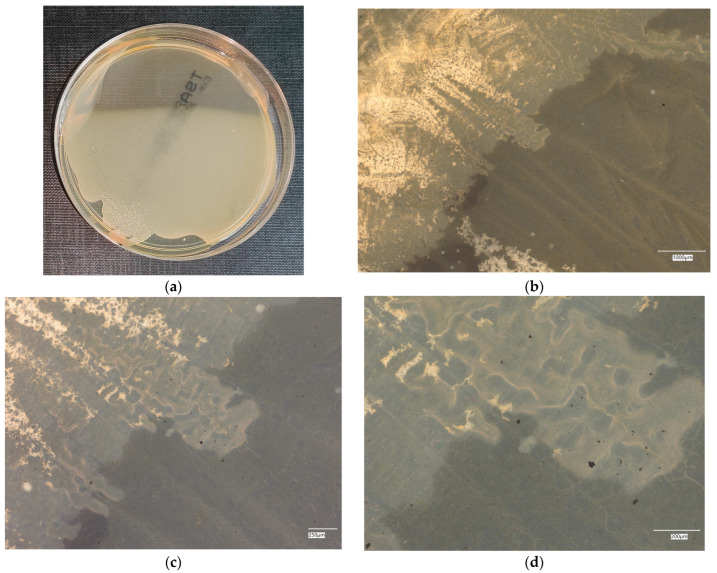
*Staphylococcus aureus* on reference plates on sample 2—geopolymer with 1% CF: (**a**) Overall photo; (**b**) Optical microscope image under magnification 40×; (**c**) Optical microscope image under magnification 100×; (**d**) Optical microscope image under magnification 200×.

**Figure 18:**
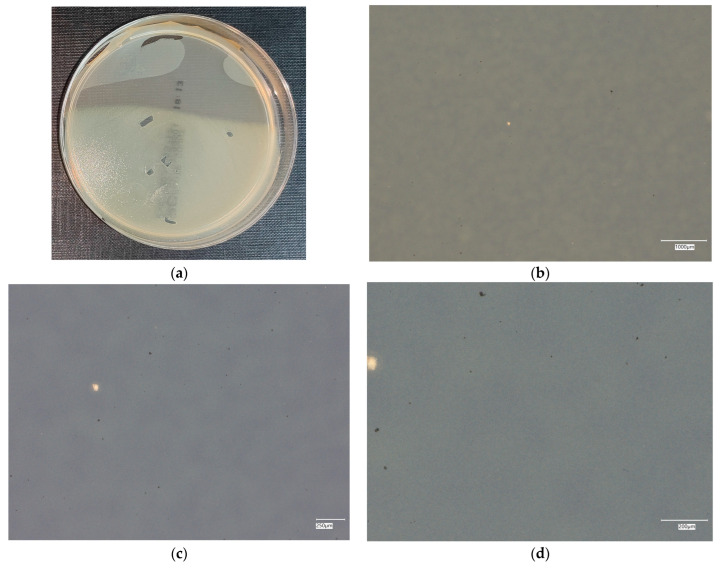
*Staphylococcus aureus* on reference plates on sample 3—geopolymer with 1% CF and 0.5% nanosilica: (**a**) Overall photo; (**b**) Optical microscope image under magnification 40×; (**c**) Optical microscope image under magnification 100×; (**d**) Optical microscope image under magnification 200×.

**Figure 19:**
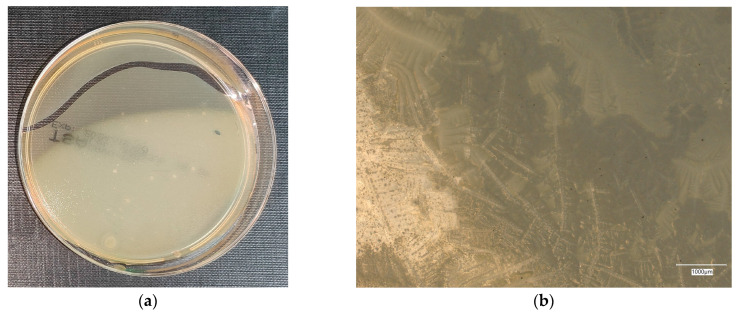

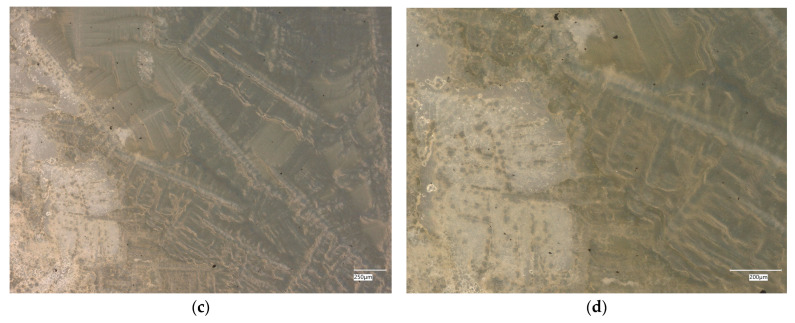
*Staphylococcus aureus* on reference plates on sample 4—geopolymer with 1% CF, 0.5% nanosilica and 0.5% antibacterial agent: (**a**) Overall photo; (**b**) Optical microscope image under magnification 40×; (**c**) Optical microscope image under magnification 100×; (**d**) Optical microscope image under magnification 200×.

**Figure 20:**
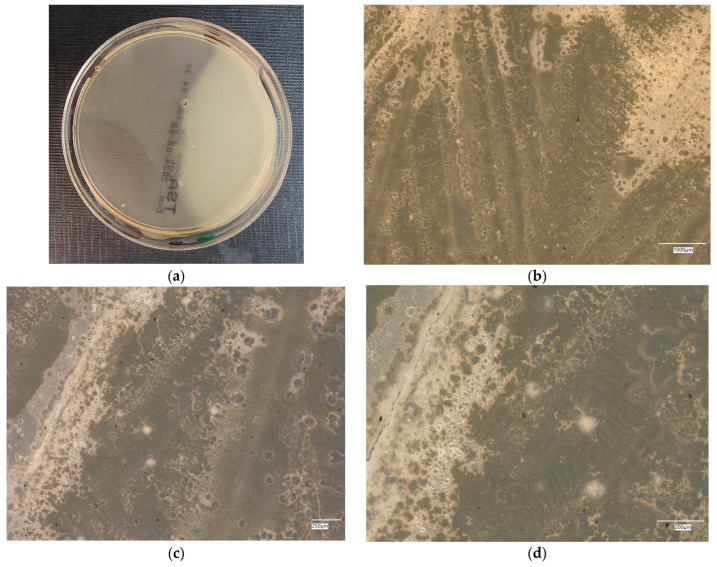
*Staphylococcus aureus* on reference plates on sample 5—geopolymer with 1% CF, 0.5% nanosilica and 1% antibacterial agent: (**a**) Overall photo; (**b**) Optical microscope image under magnification 40×; (**c**) Optical microscope image under magnification 100×; (**d**) Optical microscope image under magnification 200×.

The results of the bacterial growth are presented in Figure 16, Figure 17, Figure 18, Figure 19 and Figure 20. The following are the specifications of the plates used for the bacteria:

- Inoculum of 10–100 colony-forming units (cfu).
- Incubation conditions: up to 3 days @ 30–35 °C, aerobic.
- *E. coli* ATCC^®^ 8739™ 2–10 mm, cream colonies.
- *S. aureus* ATCC^®^ 6538™ 1–2 mm, cream shiny colonies.
- *Pseudomonas aeruginosa* ATCC^®^ 9027™ 3–8 mm, green–yellow colonies.
- *Bacillus subtilis* ATCC^®^ 6633™ 3–9 mm, cream colonies.

An analysis of the *A. aureus* culture result after incubation on reference plates shows clusters of cream-colored microorganism colonies. The largest shiny, cream-colored colony area is observed on plate no. 1, which is concerned with cement concrete. Plates with geopolymer fluid infected with microorganisms show clusters clearly aligned with the movement of the culture.

#### 3.4.4. *Staphylococcus aureus* on Chromogenic Plates

**Figure 21:**
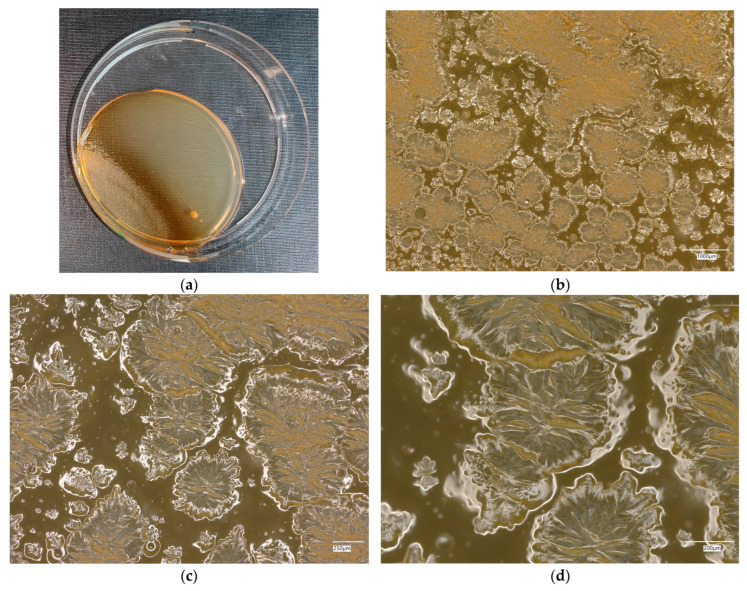
*Staphylococcus aureus* on chromatic plates on sample 1—cement material: (**a**) Overall photo; (**b**) Optical microscope image under magnification 40×; (**c**) Optical microscope image under magnification 100×; (**d**) Optical microscope image under magnification 200×.

**Figure 22:**
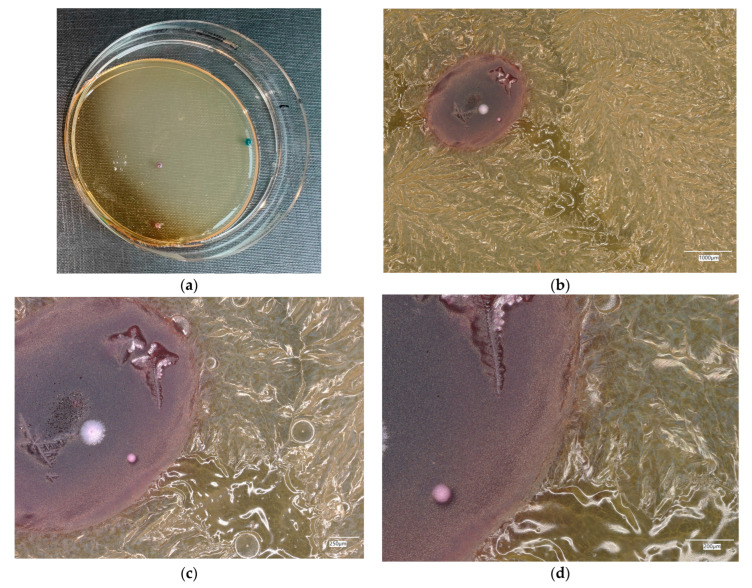
*Staphylococcus aureus* on chromatic plates on sample 2—geopolymer with 1% CF: (**a**) Overall photo; (**b**) Optical microscope image under magnification 40×; (**c**) Optical microscope image under magnification 100×; (**d**) Optical microscope image under magnification 200×.

**Figure 23:**
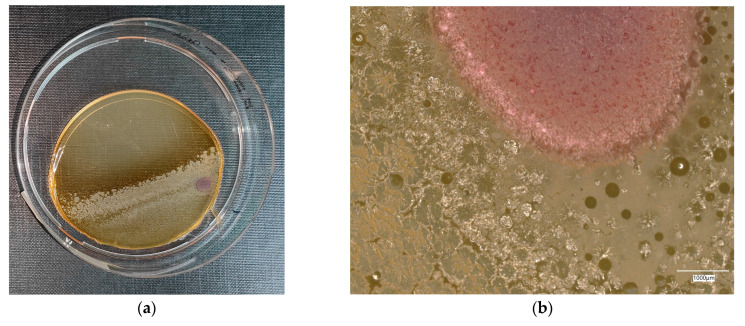

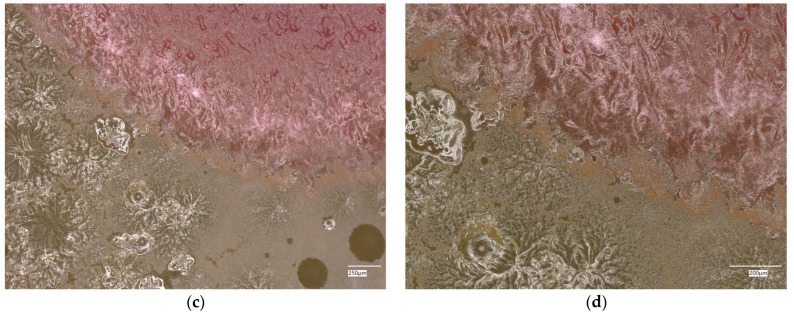
*Staphylococcus aureus* on chromatic plates on sample 3—geopolymer with 1% CF and 0.5% nanosilica: (**a**) Overall photo; (**b**) Optical microscope image under magnification 40×; (**c**) Optical microscope image under magnification 100×; (**d**) Optical microscope image under magnification 200×.

**Figure 24:**
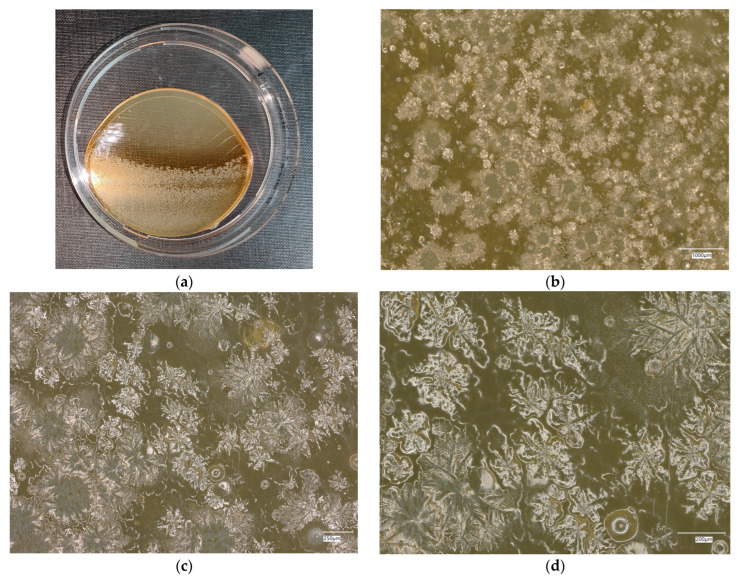
*Staphylococcus aureus* on chromatic plates on sample 4—geopolymer with 1% CF, 0.5% nanosilica and 0.5% antibacterial agent: (**a**) Overall photo; (**b**) Optical microscope image under magnification 40×; (**c**) Optical microscope image under magnification 100×; (**d**) Optical microscope image under magnification 200×.

**Figure 25:**
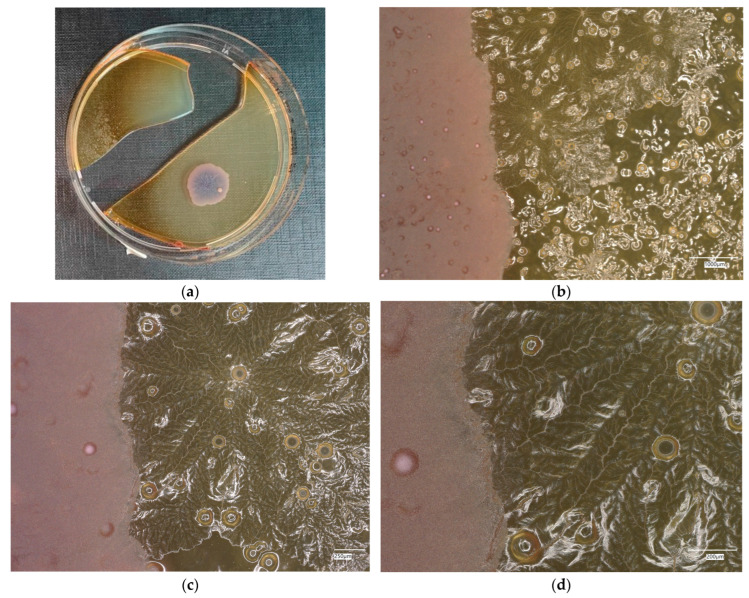
*Staphylococcus aureus* on chromatic plates on sample 5—geopolymer with 1% CF, 0.5% nanosilica and 1% antibacterial agent: (**a**) Overall photo; (**b**) Optical microscope image under magnification 40×; (**c**) Optical microscope image under magnification 100×; (**d**) Optical microscope image under magnification 200×.

The results of the bacteria growth are presented in Figure 21, Figure 22, Figure 23, Figure 24 and Figure 25. The following are the specifications of the plates used for the bacteria:

- Microbiological control of positive control growth inoculum of 50–120 colony-forming units (cfu), quantitative.
- Incubation conditions: 18–24 h @ 36 ± 1 °C, aerobic *S. aureus* ATCC^®^ 33591™ 1–2 mm, blue colonies.
- Colony counts shall be ≥50% of the control medium TSA.

The microbiological control of the chromogenic plate with the *S. aureus* bacteria shows the presence of microorganisms in all material samples. A clear presence of colonies is marked on samples related to geopolymers, taking the form of purple–blue clusters. The largest area infected with microorganisms is located on plate no. 5, with a 1% addition of an antibacterial agent, where a dozen or so new, growing clusters are observed at 100× magnification.

## 4. Discussion

### 4.1. The Discussion of the Obtained Results and Identifying Possible Mechanisms of Bacteria Growth Inhibition

The presented research has a mainly qualitative character that is determined by the method used for research with the use a bacteria strain. The results obtained for two types of the used bacteria strains, Gram-negative (*Escherichia coli*) and Gram-positive (*Staphylococcus aureus*), show similarities.

The first experiment was connected with the assessment of microorganism growth inhibition by cement and a geopolymer. In this case, the behavior of the *E. coli* was according to the expectation. The reference sample—water—clearly shows the growth of colonies similar to the cement sample. The other samples based on geopolymers inhibited the growth of the microorganism. In the case of *S. aureus* microorganisms, the experiment does not give clear results. The colony growth was observed for all samples, including those with an antibacterial agent.

The more specific data were obtained from the experiment with the evaluation of microbial multiplication in liquid with cement and geopolymer samples. In this case of reference plates, the *E. coli* and *A. aureus* microorganism cultures grow the most intensively on cement samples. The growth of the geopolymer samples was similar for all compositions. It can be assessed as less intensive than for cement, but without significant differences between particular geopolymer composites. In the case of chromatographic plates for *E. coli* colonies, the intensive growth was also observed for cement. In the case of geopolymer samples, the growth of bacteria was less intensive with increasing additives in the geopolymer composition. S. *aureus* bacteria show the presence of microorganisms in all material samples. In this case, the correlation with the amount of the additives was not clear.

The provided investigation shows the inhibition of bacterial growth, which could be caused by several phenomena (parallel mechanisms) [15]. The reaction that is delayed in time suggests that cells are not directly destroyed, but rather indicates the formation of reactive oxygen species that penetrate the cell membrane. Also, the mechanism connected with the influence of the DNA geometry and metabolism routes of the oxides should be taken into consideration [7,11]. In the short time, the supporting mechanism can also be connected with the high pH of the investigated materials [39].

### 4.2. Comparison of the Obtained Results with the Literature

The provided research shows the growth of both types of bacteria on the materials, which proves a lack of toxicity in the composition. Next, after several days, the growth was inhibited. In this case, the geopolymer samples (no. 2–5) show better efficiency than cement (no. 1). According to the geopolymer composition, it can be observed that the antibacterial additive did not always fulfill its role, and there is no significant difference between the samples with and without additives. The obtained results suggest that the material inhibits their growth over time. The obtained results have been compared to other research provided mainly for standard “on land” applications, because of a lack of previous tests dedicated to the application of geopolymers in marine or freshwater environments—Table 4.

The previous research is coherent in some points, but also shows significant differences. The main point is whether the geopolymers have antibacterial properties themselves or if special additives are required. The investigation provided by Mohsen et al. [1] suggests that geopolymers are a fertile environment for microbial development and that only proper additives can give them the required antimicrobial properties [1]. Other research shows that the geopolymer has no significant effect on bacteria [48]. Most of the research confirms the antibacterial properties of a material that can be additionally enhanced by different additives [7,19]. The literature data clearly point out that the addition of nanoparticles increases the antimicrobial properties of the material [23]. The obtained results confirmed some potential of geopolymer applications in antibacterial applications, but also clearly show that the material properties are enhanced by nanoparticles.

### 4.3. The Novelty of the Presented Study Compared with the Literature

The new element in this research was to plan this research from the point of view of application in the water environment. The provided research can be useful for the prediction of biofouling phenomena on marine and inland water infrastructure. Biofouling is a biological mechanism of the occurrence of aquatic or semi-aquatic microorganisms on a material’s surface. In the case of most applications, this phenomenon is unwanted because it causes the biocorrosion of infrastructure, for example, submerged pipelines, or increases the cost of transportation in the case of boat hulls. For some particular applications, it could be a desirable phenomenon, for example, in the design of coral reefs [49]. Currently, only a few research studies have been connected with biofouling phenomena on geopolymer materials [49,50,51]. They have been provided on modified geopolymers or covered materials. Subaer et al. [51] modified geopolymers with rGO and TiO_2_ nanoparticles and confirmed that TiO_2_ acts as a photocatalyst and an anti-biofouling material [51]. Also, the better anti-biofouling properties compared to cement were confirmed by Ly et al. [49]. Additionally, to enhance the anti-biofouling properties, the hydrogel can be used as a coating on geopolymer materials [50]. The presented research is in line with previous investigations and based on the obtained results, it can be pointed out that the development of *Escherichia coli* and *Staphylococcus aureus* was inhibited on the geopolymer materials.

### 4.4. The Limitations of This Study and Further Research Directions

The provided research on antimicrobial properties shows that geopolymer composites can find applications in the marine or freshwater infrastructure. The properties connected with inhibiting the bacteria’s growth can also be desired in different applications, including medical infrastructure, the aerospace industry and others where the presence of the bacteria is not desired. However, further research is needed to better understand the mechanism itself and provide some more advanced analysis, including the determination of the spreading speed in different conditions and the determination of the material efficiency over time. In the case of some bacterial strains, the nanoadditives seem to be a reasonable option; however, their efficiency does not work with the same efficiency for all types of microorganisms. It suggests that the mechanism in the material is complex and does not appear in only one phenomenon. It suggests that for the design of the antibacterial materials, more than one type of additive should be considered, and to protect the infrastructure, the composition with several additives should be taken into consideration.

## 5. Conclusions

The provided research shows the possibility to synthesize geopolymers with antibacterial additives, such as carbon fiber, nanosilica and antibacterial nanopowder, being a mixture of metals and oxides such as: Ag, P_2_O_5_, ZrO_2_, N_2_O, Y_2_O_3_, Al_2_O_3_, HfO_2_, TiO_2_ and others. Next, the microorganism growth using two types of colonies (Gram-positive and Gram-negative) on the material was studied. The provided research allows us to formulate the following conclusions:

- The main components of the mineralogical composition for designed geeopolymers are quartz, 55.0%, and mullite, 42.1% of the crystalline ingredients.
- The main oxide component for the geopolymer composition is SiO_2_.
- The obtained results show the growth of both types of bacteria on the materials; however, after several days, the growth was inhibited.
- An assessment of microorganism growth inhibition by the cement and geopolymers shows the better efficiency of geopolymer composites in this area (in most cases).
- It should be noted that in the case of geopolymer materials, the antibacterial additive did not always fulfill its role.
- The obtained results suggest that the material does not negatively influence the bacteria, but inhibits their growth over time.
- This effect is desired in the context of potential applications of the material on water infrastructure.

The obtained results suggest that the material does not negatively influence the bacteria, but inhibits their growth over time. These findings allow us to make the prognosis that the composition is suitable for infrastructural applications where resistance against the growth of microbes is necessary.

## Figures and Tables

**Table 1:** Sample designation.

Designation	Cement [%]	Sand [%]	Fly Ash [%]	Coal Shale from Silesia Mine [%]	Carbon Fiber [%]	Nanosilica [%]	Antibacterial Agent [%]	Solution
1	50	50	-	-	-	-	-	-
2	-	-	79	20	1	-	-	10 M
3	-	-	78.5	20	1	0.5	-	10 M
4	-	-	78	20	1	0.5	0.5	10 M
5	-	-	77.5	20	1	0.5	1	10 M

**Table 2:** Results for EDS analysis of geopolymer composite (all oxides are identified on line K).

No.	Oxide	Amount [%wt.]
1	Na_2_O	23.9 ± 0.2
2	MgO	1.4 ± 0.2
3	Al_2_O_3_	16.0 ± 0.2
4	SiO_2_	50.9 ± 0.3
5	SO_3_	2.9 ± 0.1
6	K_2_O	2.5 ± 0.1
7	CaO	2.4 ± 0.1

**Table 3:** Results of pH measurements for samples designated as 1–5 from Table 1 and additionally for reference sample “0”—water. The results were obtained after different periods of time.

Designation	Time				Average
	15 min	24 h	48 h	72 h	
1	10.6	12.45	12.40	12.51	11.99
2	11.30	12.19	12.27	12.08	11.96
3	10.62	11.65	11.90	12.03	11.55
4	11.42	12.35	12.40	12.25	12.11
5	11.66	12.51	12.53	12.47	12.29
0	-	9.26	8.76	9.05	9.02

**Table 4:** Previous research on the antibacterial properties of geopolymers.

No.	Geopolymer (Composition)	Investigated Bacteria	Results	Source
1	Metakaolin activated by sodium hydroxide and sodium silicate	*Escherichia coli*, *Pseudomonas aeruginosa*, *Staphylococcus aureus*, *Enterococcus faecalis*	Antimicrobial properties against *E. coli* bacteria Decrease in cell viability of mitochondrial activity of fibroblast murine NIH-3T3.	[41]
2	Ground-granulated blast-furnace slag, metakaolin, fly ash, lead-bearing sludge, superplasticizer, activated by sodium hydroxide solution	*Salmonella typhi*, *Bacillus cereus*	The materials show anti-microbial activity against both tested microbial strains.	[9]
3	Metakaolin, suction dust, red mud, sludge from the food supplement industry, and sludge from partially stabilized industrial waste. Activation by sodium hydroxide and sodium silicate	*Escherichia coli*, *Staphylococcus aureus*	Confirmation of activity against bacterial strain properties for designed geopolymers, especially those that contain industrial waste. The possibility of application of geopolymer coatings against nosocomial infections.	[42]
4	Ground-granulated blast-furnace slag with nanochromia activated by sodium hydroxide solution	*Escherichia coli*, *Staphylococcus aureus*	Geopolymer composites with nanochromia inhibit the growth of microbial strains.	[16]
5	Ground-granulated blast-furnace slag, red brick waste, sodium hydroxide, superplasticizer, nanoparticles (WO_3_)	*Salmonella typhi*, *Staphylococcus aureus*	The antimicrobial properties of geopolymers are correlated to the presence of nanoparticles (WO_3_).	[17]
6	Metakaolin, microparticles (silver, copper and nickel), potassium-based activator	*Escherichia coli*, *Micrococcus luteus*	There was no clearly confirmed antibacterial activity of geopolymers without additives. The microparticles enhanced the antimicrobial activity.	[43,44]
7	Ground-granulated blast-furnace slag, fly ash, sodium hydroxide, superplasticizer, meso-porous nickel oxide nanoparticles	*Salmonella typhi*, *Klebsiella pneumonia*, *Enterococcus faecalis*	The slag/fly-ash-based geopolymer is a fertile environment for microorganism development. The addition of nickel oxide nanoparticles provides antibacterial properties for the material.	[1]
8	Ground-granulated blast-furnace slag, fly ash, cement kiln dust, metakaolin, superplasticizer activated by sodium hydroxide solution	*Bacillus cereus*, *Salmonella typhi*	The prepared geopolymers show antimicrobial resistance, the efficiency was dependent on the proportion of particular ingredients.	[45]
9	Fly ash, metakaolin, nano-titanium oxide, activated by sodium hydroxide and sodium silicate	*Escherichia coli*, *Candida albicans*, *Pseudomonas aeruginosa*	Nano-titanium oxide enhances the antimicrobial properties of geopolymers.	[19]
10	Tuff, sodium water glass, potassium hydroxide	*Escherichia coli*, *Staphylococcus aureus*	Antimicrobial tests against bacteria confirmed a wide spectrum of activity of the designed geopolymer paints.	[3]
11	Ground-granulated blast-furnace and hematite nanoparticles activated by sodium hydroxide	*Escherichia coli*, *Staphylococcus aureus*	The addition of mesoporous hematite nanoparticles to the geopolymer matrix significantly enhances antimicrobial properties.	[13]
12	Natural zeolite powder, ground-granulated blast-furnace, activated by sodium silicate solution	*Escherichia coli*	The geopolymers containing the zeolite had a little less antibacterial activity.	[46]
13	Metakaolin, powder glasses from waste electric and electronic equipment, activated by sodium hydroxide and sodium silicate	*Escherichia coli*	The research shows biocide activity of geopolymers.	[47]
14	Metakaolin, waste glass, activated by sodium hydroxide and sodium silicate	*Escherichia coli*, *Enterococcus faecalis*	Geopolymers do not have a strong impact on living organisms, showing only a slightly negative effect on *E. coli* and no effect on *E. faecalis.*	[48]
15	Ground-granulated blast-furnace slag and lead-bearing sludge activated by sodium hydroxide	*Enterococcus faecalis*, *Bacillus subtillis*, *Escherichia coli*	Anti-bacterial activity increases with lead-bearing sludge content.	[7]
16	Class F fly ash, zinc oxide nano-rods, silica nanoparticles, activated by sodium hydroxide and sodium silicate	*Escherichia coli*, *Staphylococcus aureus*, *Aspergillus niger*	The proposed composition has significant anti-microbial properties.	[15]
17	Class F fly ash, colloidal nano-silica, silver nanoparticles, activated by sodium hydroxide and sodium silicate	*Escherichia coli*, *Staphylococcus aureus*	The addition of silver–silica nanocomposite improves significantly the antibacterial properties of the geopolymer mortar.	[22]

## Data Availability

The original contributions presented in this study are included in this article; further inquiries can be directed to the corresponding author.

## Abbreviations

The following abbreviations are used in this manuscript:

EDS	energy-dispersive spectrometer
SEM	scanning electron microscopy
XRD	X-ray diffraction
